# Modern Sociology

**Published:** 1896-12-05

**Authors:** 


### Modern Sociology.

### MISTAKEN IMPRESSIONS OF PRISON LIFE.—V.

The duty of the State towards criminals has been a subject of discussion by jurists and philanthropists for a very long time, and contemporary writers of the soundest judgment are still at variance respecting it. Only one principle seems to have been firmly established, that no obligation to punish the criminal exists, excepting such as is involved in the necessity of protecting the interests of the community, and that "the public welfare is the highest law." This principle is, of course, perfectly sound and true, and must be accepted as the ground-work of judicial legislation; but we cannot admit the contention of some able writers, that it is to be held the only one on which the administration of justice is to be based, and that our rulers are in no sense bound to aim at the reformation of law-breakers, or at the suppression of the growth of crime generally. On either point, and still more on both, the wise and just treatment of professional criminals, is by far the most difficult of the duties which the State has to perform in this connection. The full significance of the term "professional crime" may be illustrated by a case which came under our personal notice. A man who had been a thief from childhood quickly developed into the armed burglar, and had been all his days a law-breaker in every sense of the word. He had a family whom he brought up to follow his own trade of robbery—murder, if necessary, and every offence against society that they could by any means perpetrate. Father and children alike were in a condition of crass ignorance. They had had no education of any kind, except in vice, and had no desires beyond the supply of their daily wants and the indulgence of their lawless tastes and passions. Their history came under our notice when one, the youngest of the sons, imprisoned for a daring burglary, made no concealment of his intention to continue his habitual trade when he left its secure walls. It is for the evils involved in the existence of criminals of this description that the thoughtful writer of whom we have spoken suggests a drastic remedy, which will not, we believe, commend itself to anyone who has studied the subject from a knowledge of the interior life of the prison, and not merely from its apparent results as seen in the outer world. We give his suggestion in his own words: "The only solution of the problem will be found in the creation of what might be described as an asylum prison, to which such prisoners should be drafted after completing a term of penal servitude." He intends that their incarceration in this novel establishment shall be for life, and bases this proposal on grounds which we hold to be greatly mistaken. In the first place, he considers that if it were practically carried out, it could be done within limits much more circumscribed than would really be required. He says: "People talk as if the masses of our population were kept from crime only by its penalties, and it is a slander on the toiling, suffering poor." Of course, we gladly admit that there are vast numbers of honest poor people who will suffer bitter privation rather than commit a single serious crime, but at the same time we affirm that there is a substratum of the very lowest and most degraded classes who have no moral sense, no belief in any Divine Lawgiver or respect for human obligations, and who are socialists and revolutionaries in so far as they are able to grasp the principles which brought the Reign of Terror upon France. If the deterrent of certain punishment for crime were removed, they would rise from their lairs like wild beasts of prey, and overrun the whole country with every species of excess and violence in the seizure of other people's goods. The writer objects to sentences which merely let criminals loose again on society after longer or shorter periods; but prison asylums would have to be planted broadcast over the land if they had to be provided for all the inmates who would be sent to them from such sources as we have described. Again, the writer seems to consider temporary imprisonment as absolutely valueless in its moral effects. He believes that every criminal comes out after five years' penal servitude exactly as he goes in, mentally and morally. Some do, undoubtedly, but we deny absolutely that this is always the case. The agencies within the prison for the spiritual and moral elevation of the criminal are neither worthless nor effete, and we have known instances, even among professional criminals, who have proved the good results of the educational care bestowed upon them during their incarceration, by steady reformation on their release. Nor must one great fact in the punishment of wrongdoers, as at present enforced, be ignored—that it is the only effective education as to the difference between right and wrong, as to what is wickedness or what is not, which the most degraded classes ever practically receive. We have come across many men and women who see no reason why they should not indulge in theft or violence, or any other iniquity if it suits them to do so. Their own vicious inclinations would be their sole law and guide, were it not that they know the Legislature holds certain actions to be criminal and deserving of punishment, and thus some enlightenment as to the distinction between good and evil is forced upon them. While, however, we do not at all agree with the writer we have quoted, as to the uselessness of the system of temporary imprisonments at present in force, we are fully alive to the necessity of some measure being adopted for the wise disposal of criminals after they leave the gaol, both with the view of relieving society from the evils of their presence and of giving to themselves a chance of earning an honest living. It seems to us that both these ends might be attained by a Government system of emigration, totally different from the old plan of transportation, which has been wisely abolished. If discharged prisoners could be sent out as free men to those vast tracts of unreclaimed land in the colonies, which might be made valuable by cultivation, they would find themselves in a position where they would be compelled to use manual labour for their own support, and where neither the incentives nor the means for a career of crime would any longer exist for them. This may not be a practicable scheme, and we give the suggestion for what it is worth; but we are confident that no expert in such matters would approve of the idea of asylum prisons if any attempt is made to bring that plan under the notice of our legislators.

